# Isolation and cultivation of candidate phyla radiation *Saccharibacteria* (TM7) bacteria in coculture with bacterial hosts

**DOI:** 10.1080/20002297.2020.1814666

**Published:** 2020-09-06

**Authors:** Pallavi P. Murugkar, Andrew J. Collins, Tsute Chen, Floyd E. Dewhirst

**Affiliations:** a Department of Microbiology, The Forsyth Institute, Cambridge, MA, USA; b Department of Oral Medicine, Infection and Immunity, Harvard School of Dental Medicine, Boston, MA, USA

**Keywords:** Saccharibacteria, TM7, cultivation, phylogeny, genomics, candidate phylum radiation

## Abstract

**Background:**

The vast majority of bacteria on earth have not yet been cultivated. There are many bacterial phyla with no cultivated examples including most members of the Candidate Phylum Radiation with the exception of human oral isolates from the phylum Saccharibacteria.

**Aims:**

The aims of this research were to develop reproducible methods and validate approaches for the cultivation of human oral Saccharibacteria and to identify the conceptual pitfalls that delayed isolation of these bacteria for 20 years after their discovery.

**Methods:**

Oral samples were dispersed and passed through 0.2 µm membrane filters. The ultrasmall saccharibacterial cells in the filtrate were pelleted, inoculated into broth cultures of potential bacterial host cells and passaged into fresh medium every 2–3 days.

**Results:**

Thirty-two isolates representing four species of Saccharibacteria were isolated in stable coculture with three species of host bacteria from the phylum *Actinobacteria*. Complete genome sequences were obtained for 16 isolates.

**Conclusions:**

Human oral Saccharibacteria are obligate bacterial parasites that can be stably passaged in coculture with specific species of host bacteria. Isolating these important members of the human oral microbiome, and many natural environments, requires abandoning many of Koch’s concepts and methods and embracing novel microbiological approaches.

## Introduction

Only a small fraction of earth’s microbes has been cultured and a majority of these microbes are from a few well-known phyla including the *Proteobacteria, Firmicutes, Actinobacteria, Fusobacteria*, and *Bacteroidetes*. However, microbiologists using molecular methods have shown that there exists a vast microbial diversity beyond that in phyla with no cultured representatives. The uncultured microbes have been referred to as ‘microbial dark matter’, as we know little about them. Microbiologists from many countries have worked diligently to isolate and culture bacteria from the often-ubiquitous uncultured phyla, such as TM7, a phylum first recognized in the 1990’s [1,2]. However, microbiologists were uniformly unsuccessful in these efforts until He and colleagues isolated a human oral strain, TM7x, in binary coculture with a strain of *Actinomyces odontolyticus* [3]. Here, we report building on He’s success to develop an approach that has allowed the isolation and cultivation of 32 TM7 (Saccharibacteria) strains representing four distinct species.

### The uncultivated majority

The development of molecular techniques to sequence 16S rRNA genes in the late 1980s and early 1990s led to the identification of a vast microbial diversity beyond that known from studying cultured microbes [4–8]. Many of the sequences obtained branched away from known phyla in 16S rRNA-based phylogenetic trees and were recognized as novel bacterial divisions and given obscure names such as TM7 [1], OP11 [9], SR1 [10], and GN02 [11] reflecting sample origins. These early 16S rRNA studies and those that followed provided a basic glimpse of the phylogenetic diversity of prokaryotic life based on a single gene but gave no insight into the metabolic and genomic potential of these candidate phyla.

### Microbial dark matter

Banfield’s group studying the microbes in acid mine drainage was the first to show that genomic sequencing could be applied to bacterial communities [12,13]. From the early efforts in community genomics, now more commonly called metagenomics, began our understanding of microbial metabolic potential of the uncultured world. Marcy *et al*. were among the first to use the term ‘dark matter’ to refer to microbes from uncultivated linages in a landmark paper on genetic analysis of TM7 bacteria from the human mouth by single-cell manipulation and subsequent sequencing [14]. The term ‘microbial dark matter’ was introduced by Rinke *et al*. in one of the first major papers describing the phylogeny and coding potential of bacterial taxa from 29 uncultivated phyla using single-cell sequencing [15]. Several major papers describing genomes of uncultured organisms were published around 2013 [15–19] providing an initial glimpse of the breadth of microbial genomic potential. With the ability to obtain complete genome sequences for uncultured organisms, investigators began giving ‘*Candidatus*’ names to the uncultured phyla. The TM7 phylum was named ‘*Candidatus Saccharibacteria*’ based on the complete genome sequence of ‘*Candidatus Saccharimonas aalborgensis*’ by Albertsen *et al*. [19]. With the initiation of *Candidatus* naming, the ‘uncultured phyla’ became the ‘candidate phyla’. Twenty-six phyla were given names by Brown *et al*. [20].

### Candidate Phyla Radiation

Banfield’s group recognized that a subset of the candidate phyla had unusual biology and was a bacterial clade that represented more than 15% of life on earth [20]. They named this clade the Candidate Phyla Radiation (CPR). Hug *et al*., presented an updated, and all-inclusive, tree of life based on analysis of 16 concatenated conserved ribosomal proteins, which showed the CPR group as distinct from other bacteria, archaea, and eukaryotes [21,22]. Based on recovery of approximately 8,000 metagenome-assembled genomes, Parks *et al*. presented a tree of life based on 120 conserved proteins [23]. They observed, however, that the size and diversity of the CPR differs with choice of protein marker set. While the number of phyla within the CPR and the appropriate criteria for delineation of super-phyla, phyla and class are a subject of debate, the diversity and importance of CPR is indisputable.

### CPR bacteria have small genomes

As the complete, or nearly complete, genomes of CPR organisms were determined; it became evident that most were approximately 1 Mbp or less in size. For example, Kantor *et al*. [18] determined the genomes of SR1 (assembly RAAC1), WWE3 (RAAC2), TM7 (RAAC3), and OD1 (RAAC4) were between 0.7 and 1.17 Mbp. Albertsen *et al*., found the TM7 metagenomes assembled for four TM7s from an activated sludge bioreactor were between 0.9 and 1.0 Mbp [19]. Campbell *et al*. found SR1 (assembly OR1) plus other assemblies gave an estimated size of 1.1 Mbp [17]. Genomes for many additional CPR phyla have been obtained since 2012 and support the generalization that CPR taxa genomes mostly smaller than 1 Mbp, and the remaining less than 1.5 Mbp [20,24]. Genome analyses indicate that all CPR bacteria have limited biosynthetic ability and are therefore highly auxotrophic [15,16,18,19]. It was noted by several authors that organisms with similar small genome size and limited synthetic capability are usually obligate symbionts or parasites of other organisms [18,20,24].

Obtaining a bacterial isolate is the key step for phenotypic characterization, genetic manipulation and commercial exploitation. While much can be learned from an organism’s metagenome-assembled genome, a complete microbiological understanding requires being able to work with and experiment on an isolate at the bench. Therefore, the primary goal of the research reported here was to develop an approach for the isolation and culture of novel human oral Saccharibacteria in coculture with their hosts based on the pioneering work of He and coworkers. A second goal was to validate the newly developed isolation and cultivation methods on larger sets of human subjects and to obtain basic phenotypic and genomic information on the strains of taxa isolated. A third goal of the research was to identify conceptual pitfalls that for many years prevented isolation of any CPR organisms. This report discusses the approaches developed and how they may be applied to cultivation of Saccharibacteria from other host-associated microbiomes, from the environment, and possibly other phyla of CPR bacteria.

## Materials and methods

### Clinical methods

#### Subjects

The subjects in this study were recruited specifically to provide oral samples for attempts to culture as yet uncultured human oral bacteria. Inclusion criterion was simply age 18 years and older. Individuals who required antibiotic premedication prior to subgingival scaling were excluded (such as subjects with heart valve issues or artificial joints). Gender balance and racial diversity were sought, the study had IRB approval (#14-10) and all subjects signed Informed Consent. Some subjects were additionally consented to self-sample. There were two sets of subjects in the Saccharibacteria isolation studies reported here. Set 1 was comprised of 35 subjects who were initially sampled at 9 sites in their oral cavities and their site microbial compositions determined by Illumina microbiome sequencing of the 16S rDNA V1-V3 region. Based on Saccharibacteria taxa-subject-site abundance, subjects with high levels were identified and resampled one or more times for Saccharibacteria isolation and cultivation. Set 2 was comprised of 14 subjects who were recruited from attendees at the Forsyth Symposium on ‘Uncultivable Bacteria’, held October 11–12, 2018, Cambridge, Massachusetts. Following informed consent, these subjects self-sampled supra-gingival plaque. Abundance of Saccharibacteria in these subjects’ mouths was unknown at time of sampling, as they had not been screened by Illumina microbiome sequencing.

#### Sampling

Baseline samples for all Set 1 subjects were obtained as follows. Sub- and supra-gingival samples were collected using Gracey curettes (Hu-Friedy Mfg, Co, Inc., Chicago, IL). Soft tissue sites (keratinized gingivae, tongue, cheek, palate, throat and tonsils) were sampled using cytology brushes (Moore Medical LLC, Chicago, IL). Unstimulated saliva was collected by having the subject drool into a sterile 15 mL screw cap centrifuge tube (Corning, Corning, NY). Samples were transferred from curette or brush into 1 mL of 10 mM Tris-HCl buffer pH 7.2 (Tris Buffer).

Samples for obtaining isolates for subject Set 1 were nearly all obtained in the Forsyth Clinic using the procedure described above. A subset of subjected in Set 1 were consented for self-sampling in the laboratory. These samples were obtained using a sterile toothpick, pipette tip or cytology brush. The toothpick, pipette tip or brush were run along the gum line and the supra gingival plaque was collected. The plaque was dislodged from the collection device by swirling in 1 mL of sterile Maximum Recovery Diluent (MRD; Peptone 1.0 g/L, Sodium Chloride 8.5 g/L, Final pH: 7.0 ± 0.2 at 25°C) in a sterile 1.5 mL centrifuge tube.

Sampling for subject Set 2 was performed by subjects brushing the buccal margins of teeth and gums with a cytology brush. The material on the brush was dispersed by twirling in 5 mL of MRD in 15 mL sterile screw cap centrifuge tube.

### Microbiological methods

#### Media

Media for plate culture included Bacto™ Brain Heart Infusion Agar (BHI-agar), Bacto™ Trypticase Soy Agar (TSA; Becton, Dickinson and Co., Sparks, MD) or Fastidious Anaerobe Agar (FAA; Acumedia Manufactures, Inc., Lansing, MI) or TSA and BHI (1:1) with Bacto™ Yeast Extract (Y) 10 g/L (TSBY-agar). Sheep’s blood (Northeast Laboratory Services, Winslow, ME), 5%, was routinely added to TSBY-agar plates. Hemin (H) (5 mg/L), the vitamin K precursor 1,4-dihydroxy-2-naphthoic acid (DHNA) 50 μg/L and nicotinamide adenine dinucleotide (NAD^+^ or Factor V) 1 mg/L (chemicals from Sigma-Aldrich, Milwaukee, WI) were added as noted for culture of some fastidious hosts.

Media for broth culture of saccharibacteria and hosts was Bacto™ Brain Heart Infusion Broth (BHI-broth) for *Actinomyces* spp. or TSBY-broth for *Arachnia* spp. For initial isolations of AC001 and PM004, TSBY-broth was mixed 1:1 with Gibco™ RPMI 1640 (ThermoFisher Scientific, USA).

#### Culture conditions

Anaerobic culture was performed in a Coy Anaerobic Chamber (Grass Lake, MI) at 37°C with a 5% H_2_/10% CO_2_/85% N_2_ atmosphere. Microaerophilic culture was performed in a Coy Hypoxic Chamber at 37°C with a 2% O_2_/5% CO_2_/93% N_2_ atmosphere. Aerobic culture was performed in a 37°C warm room in air.

#### Strains used as potential hosts

Strains from the Forsyth Human Oral Microbe Collection were revived on suitable medium and conditions (see Table 1). Revived strains were streaked for purity and selected colonies validated by 16S rRNA sequencing. Validated strains were maintained by plate passage every 2–5 days. Prior to use as a potential host for Saccharibacteria coculture in broth, the strain was passaged in 2.2 mL of broth, by 1:11 dilution (0.2 mL added to 2.0 mL) approximately every 2 days, for at least two passages to allow cells to adapt to broth vs plate culture.

**Table 1.** Potential host species for coculture of Saccharibacteria.

Study	Phylum	Species name	Taxon	Strain number	Medium	Atmosphere	Accession No.	Seccessful coculture
1	Actinobacteria	Actinomyces graevenitzii *	HMT-618	F0530	BHI	Anaerobic	AWSC00000000	No
1	Actinobacteria	*Actinomyces johnsonii*	HMT-849	F0510	BHI	Anaerobic	AWSD00000000	No
1	Actinobacteria	Arachnia propionica *	HMT-739	F0230a	TSBY	Anaerobic	CP002734	HMT-488, HMT-955
1	Firmicutes	*Catonella morbi*	HMT-165	ATCC 51,271	TSBY	Anaerobic	ACIL00000000	No
1	Actinobacteria	*Corynebacterium matruchotii*	HMT-666	ATCC 33,806	BHI	Anaerobic	ACEB00000000	No
1	Firmicutes	*Lachnoanaerobaculum saburreum*	HMT-494	F0468	TSBY	Anaerobic	AJGH01000000	No
1	Fusobacteria	*Leptotrichia wadei*	HMT-222	F0279	TSBY	Anaerobic	AWVM00000000	No
1	Actinobacteria	*Olsenella sp.*	HMT-807	F0195	TSBY	Anaerobic	CP012069	No
1	Firmicutes	*Scardovia wiggsiae*	HMT-195	F0424	TSBY	Anaerobic	AGZS00000000	No
1	Firmicutes	*Slackia exigua*	HMT-602	ATCC 700,122	TSBY	Anaerobic	ACUX00000000	No
1	Firmicutes	Veillonella atypica *	HMT-524	ATCC 17,744	TSBY	Anaerobic	AMEX00000000	No
2	Actinobacteria	Arachnia propionica *	HMT-739	F0700	TSBY	Anaerobic	CP040007	HMT-488, HMT-955
3	Actinobacteria	Arachnia propionica *	HMT-739	F0700	TSBY	Anaerobic	CP040007	HMT-488, HMT-955
3	Actinobacteria	Actinomyces sp. *	HMT-171	F0337	BHI	Anaerobic	AECW00000000	HMT-952
3	Actinobacteria	Schaalia meyeri *	HMT-671	W712	BHI	Anaerobic	CP012072	No
4	Actinobacteria	*Schaalia odontolytica*	HMT-701	F0309	BHI	Microaerophilic	ACYT00000000	HMT-952
4	Actinobacteria	*Actinomyces sp.*	HMT-897	F0631	BHI	Microaerophilic	CP027236	HMT-349

Species followed by asterisk were first used in experiments we reported previously [25].

#### Saccharibacteria isolation procedure

Clinical samples were disrupted by vigorous vortexing in 1 mL of sterile MRD and then diluted with 9 mL additional MRD. For filtration, 47 mm 0.2-micron track-etched polycarbonate membrane filters (Isopore, MilliporeSigma, Burlington, MA) were routinely used in 47 mm Swin-Lok filter holders (Whatman, GE Healthcare, Pittsburg, PA). The assembled filters were wrapped in foil and sterilized by autoclaving prior to use. The sterile filter apparatus was flushed with 10 mL of sterile MRD via syringe to wet the membrane, wash out any preservatives and ensure there were no leaks in the assembly. The diluted clinical sample was then passed through the filter using a 10 mL syringe and the flow-through was collected in a sterilized 26.3 mL ultracentrifuge bottle (Assembly 355,618, Beckman Coulter). The filter was washed with another 10 mL of MRD and this flow-through was combined with the sample flow-through in the same bottle. Collected samples were kept on ice (< 30 min) until cells were concentrated by ultracentrifugation, using a Ti-70 rotor in a Beckman Coulter (Brea, CA), Optima L-100 centrifuge. Filtered samples were centrifuged at 60,000 x g for 1 h at 4°C. After pouring off the supernatant, the pellet (usually invisible) was resuspended in 1 mL of MRD diluent for culturing or TES for DNA extraction (see Molecular methods). For culture, the re-suspended cells were divided into between 1 and 10 aliquots depending on experiment and were added to 2 mL broth cultures of containing potential host bacteria. The potential cocultures were passaged 5 times and then tested for Saccharibacteria viability/infection as described below.

#### Saccharibacteria-host coculture and passage

Saccharibacteria-host cocultures were passaged in broth, not on plates (see Discussion for explanation of reasons). Stable cocultures, where Saccharibacteria killed only a limited fraction of their host bacteria, were passaged in 2.2 mL broth cultures by 1:11 dilution into fresh broth approximately every 2 days. Unstable cocultures, where Saccharibacteria killed essentially all of their host bacteria in 2 days, were passaged by 1:11 dilution into fresh broth containing fresh host (1:11 dilution of ongoing host broth culture).

#### Culture storage methods

To prepare cocultured cells for storage, a 2 mL culture was expanded to 10 mL using appropriate medium and added host cells if normally required for passage. After 2 d growth, the culture was centrifuged at 7,500 x g for 10 minutes to pellet Saccharibacteria attached to host cells. The supernatant was discarded. ***Storing in DMSO***: Pelleted cells were re-suspended in 5 mL broth medium. DMSO was added to achieve a final concentration of 5%. Five hundred µL aliquots of the cell suspension were pipetted into 1 mL freezer tubes and stored at −80°C for future use.

***Storage in Glycerol***: Pelleted cells were re-suspended in 4 mL broth media. One mL of glycerol was added for a final concentration of 20% (v:v). Five hundred µL aliquots of the cell suspension was pipetted into 1 mL freezer tubes and stored at −80°C for future use. Note that glycerol storage cannot be used with *Arachnia propionica* (see Results: Saccharibacteria stability, storage and revival).

#### Revival methods

The entire content of a culture stock vial, 0.5 mL, was added to 4.5 mL of fresh broth medium (containing fresh host cells if appropriate) and passaged as described above. Viability was determined as described below.

#### Saccharibacteria viability/infection testing

The presence of Saccharibacteria in a sample or culture was determined by PCR with specific Saccharibacteria primers (described in PCR molecular methods). When testing viability after revival, environmental challenge, or infection of a potential host, Saccharibacteria DNA from dead or non-dividing cells was expected to be present and to potentially give false positive PCR results, so the criteria for viability/infection was PCR positive results after 5 passages (1:11 dilution each passage and a total 1:161,051 or 5 log dilution).

#### Saccharibacteria-host coculture purity check

Normally in microbiology, culture purity is examined by streaking for isolation on an agar plate and examining colonies. When streaking a Saccharibacteria-host coculture on plates, we have found that only uninfected host cells divide to form colonies, as infected cells appear not to divide (see Discussion). Colony and microscopic morphology, Gram stain and 16S rRNA sequencing were used to detect bacterial contamination. However, microbiome analysis by 16S rRNA sequencing of cultures is considered the gold standard of culture analysis as it can detect low-level contamination of the binary coculture with a third or additional bacterial species.

#### Contaminated culture cleanup

Cocultures of Saccharibacteria and their hosts found to be contaminated with one or more additional bacteria were purified by one or more rounds of filtration, ultracentrifugation, and inoculation into broth culture with fresh host. The newly established cocultures were re-checked to validate purity.

If filtration and reinfection failed to produce an uncontaminated binary broth culture, the broth was plated on agar medium. As discussed further in the Discussion, plating primarily yield colonies of uninfected host cells, rare colonies with infected host (1 in 30 to 1 in 100 colonies) and colonies of contaminating bacteria if present. Fifty to 100 colonies were picked and inoculated into broth. Both the colonies and the resulting broth cultures were screened by PCR for presence of Saccharibacteria. The Saccharibacteria positive cultures were then validated as describe above and rechecked for contamination.

#### Scanning electron microscopy

Micrographs were obtained for Saccharibacteria-host cocultures using methods described previously [25].

### Molecular methods

#### 16S rRNA sequencing

Bacterial strains were routinely identified to species by full 6-primer 16S rRNA sequencing as described previously [26]. The electropherograms were examined for double reads and other signs that the cultures were contaminated.

#### Saccharibacteria PCR identification

Saccharibacteria were detected in clinical samples and cultures by PCR using primers 1–4 given in Table 2. These primers start with the word Identification in column titled Use. Routine PCR was carried using GoTaq Green MasterMix (Promega, Madison, WI). One µL of each culture or purified DNA solution was used as template in a reaction containing 12.5 µL of GoTaq MasterMix, 9 µL of water, 1 µL of 25 mM MgCl_2_, 0.75 µL of each specific primer (20 µM). Thermocycling conditions were 95°C for 5 min, followed by 30 cycles of 95°C denaturation for 30 s, 60°C annealing for 30 s, 72°C extension for 1 min per kb, followed by a final extension for 2 min. PCR products were visualized on a 1% agarose gel using SYBR Safe gel stain (ThermoFisher, Waltham, MA).

**Table 2.** Primers used for PCR and sequencing.

Number	Primer description	Primer	Sequence (5ʹ to 3ʹ)	Primer position	Source	Use
1	TM7 16S rRNA 580 Fwd	AI71	AYTGGGCGTAAAGAGTTGC	563–580^a^	Reference^g^	Identification 600 base 16S rRNA fragment PCR most taxa
2	TM7 16S rRNA 1177 Rev	AI72	GACCTGACATCATCCCCTCCTTCC	1177–1200^a^	Reference^h^	Identification 600 base 16S rRNA fragment PCR most taxa
3	TM7 16S rRNA 33 Fwd	AJ02	ATCCTGGCTCAGGATKAA	16–33^a^	This paper	Identification full 1500 base 16S rRNA PCR most taxa
4	TM7 16S rRNA 1524 Rev	AI85	AAGGAGGTAATCCATCCG	1524–1541^a^	This paper	Identification full 1500 base 16S rRNA PCR most taxa
5	TM7 Fingerprint 16S rRNA 1291 Fwd	AI73	AGCAAATCRCAYCAAARC	1275–1291^a^	This paper	Fingerprint region 16S rRNA to tRNA PCR
6	TM7 Fingerprint tRNA Ala 30 Rev	AI78	ACCCCCTGCTTGCAAAGCA	30–48^b^	This paper	Fingerprint region 16S rRNA to tRNA PCR
7	TM7 Fingerprint tRNA Ile 30 Rev	AI79	ACCTCGTCATTATCAGTGA	30–48^b^	This paper	Fingerprint region 16S rRNA to tRNA PCR
8	TM7 Fingerprint tRNA Val 30 Rev	AI80	CCCTCTCGGTGTAAACGA	30–47^b^	This paper	Fingerprint region 16S rRNA to tRNA PCR
9	TM7 Fingerprint tRNA Ala 42 Fwd	AI81	GCACCTGCTTTGCAAGCA	25–42^b^	This paper	Fingerprint region tRNA to 23S rRNA PCR
10	TM7 Fingerprint tRNA Ile 42 Fwd	AI82	GCGCGTCACTGATAATGA	25–42^b^	This paper	Fingerprint region tRNA to 23S rRNA PCR
11	TM7 Fingerprint tRNA Val 42 Fwd	AI83	GCATCTCGTTTACACCGA	25–42^b^	This paper	Fingerprint region tRNA to 23S rRNA PCR
12	TM7 23S rRNA 53 Rev	AJ26	GCAGTCTTCCACGTCCTT	53–70 ^c^	This paper	Fingerprint region tRNA to 23S rRNA PCR
13	TM7 23S rRNA 192 Rev	AJ27	CTACTAAGATGTTTCAGTTCA	192–212 ^c^	This paper	Fingerprint region tRNA to 23S rRNA PCR
14	TM7 23S rRNA 639 Rev	AJ28	CGGGGTTCTTTTCACCTT	639–656 ^c^	This paper	Fingerprint region tRNA to 23S rRNA PCR
15	AC001 Fingerprint RNA methyltransferase A Fwd	AJ06	GCGGAACTTGGTGAAGA	565,869–565885^d^	This paper	Differentiate Saccharibacteria HMT-955 strains AC001 and HB001
16	AC001 Fingerprint RNA methyltransferase A Rev	AJ07	CGTTAGCTTTACTAATACCCA	565,285–565303^d^	This paper	Differentiate Saccharibacteria HMT-955 strains AC001 and HB001
17	HB001 Fingerprint RNA methyltransferase A Fwd	AJ08	GCGGAGCTGAGTAAGGA	within gene^e^	This paper	Differentiate Saccharibacteria HMT-955 strains AC001 and HB001
18	HB001 Fingerprint RNA methyltransferase A Rev	AJ09	CAGCATCTTTACTGATAGCTA	within gene^e^	This paper	Differentiate Saccharibacteria HMT-955 strains AC001 and HB001
19	*Arachnia propionica* Fingerprint Fwd	AI86	GCTGAGCGTAACATGAGAT	16,066–16,084 ^f^	This paper	Differentiate *Arachnia propionica* strains F0230 and F0700
20	*Arachnia propionica* Fingerprint Rev	AI87	CCATGTACTGCAAGGAATGT	15,631–15,650 ^f^	This paper	Differentiate *Arachnia propionica* strains F0230 and F0700
21	TM7 16S rRNA HMT-488-plus but not HMT-955 Fwd	AJ30	TTCCACAATGGGCGAAAG	367–384^a^	This paper	Differentiate Saccharibacteria species HMT-488 from HMT-955
22	TM7 16S rRNA HMT-488-plus but not HMT-955 Rev	AJ31	CGGGGCAGTCCAAGTA	1150–1165^a^	This paper	Differentiate Saccharibacteria species HMT-488 from HMT-955
23	TM7 16S rRNA HMT-955-plus but not HMT-488 Fwd	AJ32	TTCCACAATGGGGGCAAC	367–384^a^	This paper	Differentiate Saccharibacteria species HMT-488 from HMT-955
24	TM7 16S rRNA HMT-955-plus but not HMT-488 Rev	AJ33	CCGGGGCAGTCTGAATA	1150–1166^a^	This paper	Differentiate Saccharibacteria species HMT-488 from HMT-955

^a^Position in 16S rRNA as aligned to *E. coli*,^b^position in tRNA, ^c^position in 23S rRNA alignment for Saccharibacteria, ^d^position in genome CP040003.1, ^e^waiting GenBank annotation, ^f^position in genome CP040007.1, ^g^Hugenholtz et al 2001, and ^h^Brining et al 2003.

#### Fingerprinting strains

All Saccharibacteria isolates were initially identified and fingerprinted by sequencing the rRNA operon from a 9–27 forward 16S rRNA primer to the third tRNA (usually tRNA Val) located between 16S and 23S rRNA genes (Table 2, primers 5–14). Specific primer pairs were designed to differentiate strains of species whose sequences differed by less than two bases over the 16S rRNA – tRNA region (Table 2, primers 15–24). Upon complete genome sequencing, the genomes were aligned, and regions of DNA sequence difference were identified. Two primer pairs about 500 bases apart in the mutual alignment were selected where each of the 4 primers was specific to its target. Alternately, a single primer pair identical for both strains, but flanking a region of 7–10 base differences between strains over a span of 500 bases was identified. Fingerprinting primers for host bacterial strains of each species were also designed from comparison of their whole genome sequences as described for Saccharibacteria.

#### Genome sequencing

Cocultures of Saccharibacteria and their hosts were passaged in broth culture and expanded from 2.2 mL to 200 mL volumes. While 0.2-micron filters were used for filtration during isolation, 0.4-micron filters were adequate to separate Saccharibacteria from their large *Actinomyces, Arachnia* or *Schaalia* hosts. The filtrate was concentrated by ultracentrifugation as described above. DNA was isolated using the following modifications of the MasterPure Gram-positive DNA purification kit protocol (Lucigen, Middletown, WI). Briefly, TES buffer (20 mM Tris-HCl, 50 mM EDTA, pH 8.0) was used in place of TE buffer. After lysozyme treatment, but prior to proteinase K treatment, samples were bead-beaten for 2 × 20 seconds with a 5-minute rest on ice in between treatments using a Fast Prep-24 (MP Biomedical). The remainder of the protocol followed the manufacturer’s instructions. Genome sequencing was performed using a PacBio instrument (Menlo Park, CA). Sequencing was performed at either Johns Hopkins University (PacBio RS II), or at the Forsyth Institute (Sequel System). Assembly was performed using HGAP. Sequences were deposited with and annotated by the NCBI Prokaryotic Genome Annotation Pipeline. Additional sequencing details have been reported for Saccharibacteria bacterium HMT-488 strain AC001 [27] and Saccharibacteria bacterium HMT-955 strain PM004 [28]. After completion of several Saccharibacteria genomes using purified Saccharibacteria DNA, we discovered that if DNA was prepared directly from the coculture, the genomes of the host and the Saccharibacteria were both easily obtained together by PacBio sequencing and both genomes assembled into single individual contigs. This second method was used to produce CM and FS series genomes and as much as 10 μg DNA could be produced from cocultures expanded to a much smaller volume of 30–40 ml.

#### Species and strain sequence comparisons

Phylogenetic trees were generated for comparison of strains and species using 16S rRNA sequences, sets of conserved proteins from genome sequencing, and 79 amino acid proteins (79 aa) found in the ribosomal RNA operons.

Full 16S rRNA sequences were aligned in RNA, a program that holds the alignment of all reference sequences at HOMD [29,30]. The aligned sequences were analyzed in MEGA X [31] using the neighbor joining program [32].

A concatenated protein tree was generated in Anvi’o [33] using the methods described by Shaiber *et al*. [34] with minor modifications. The following ribosomal proteins were used: L1, L2, L3, L4, L5, L6, L13, L14, L16, L18p, L19, L21p, L22, L23, L29, S2, S3, S6, S7, S8, S9, S11, S12_S23, S13, S15, S17 and S19. A maximum likelihood phylogenetic tree was computed using IQ-TREE [35] with the WAG general matrix model [36] and 1000 bootstrap replicates. The output treed was edited in MEGA X [31]. Average nucleotide identity (ANI) was computed using Anvi’o ‘anvi-compute-genome-similarity’ that uses blastn+ [37].

The 79 aa protein tree was generated from an alignment of the protein sequences using neighbor joining method in MEGA X [31]. Evolutionary distances were computed using the Poisson correction method [38]. The protein sequences were obtained either from the completed genome of a strain, or by sequencing the ribosomal RNA fingerprint region as described above.

#### Species and strain definitions

The human oral microbiome database was established in 2008 to provide a consistent taxonomic framework for named, unnamed and uncultured oral bacteria based on 16S rRNA phylogenetic analysis [30,39,40]. Each named species or unnamed taxonomic group (98.5% similarity for full 1,500 base sequences) was assigned a Human Microbial Taxon number such as HMT-123. The provisional naming scheme allows differentiation of *Actinomyces sp*. HMT-169 from other unnamed *Actinomyces spp*. such as *Actinomyces sp*. HMT-448. A strain is indicated by adding a strain number such as *Actinomyces sp*. HMT-169 strain F0496. These designations for species and strain will be used throughout this report. As whole genome information is now available for the majority of oral taxa, HOMD is using whole genome comparisons to refine taxon definitions, particularly in cases where 16S rRNA phylogeny is ambiguous.

## Results

### Isolation of Saccharibacteria species and strains

The primary goal of this research was to develop an approach that would allow isolation of novel human oral Saccharibacteria species and strains. Our initial approach was based on three observations and one inference from previous work. First, Saccharibacteria were obligate parasites that require a host [3]. Second, Saccharibacteria cells could pass through 0.2-micron filters and be separated from most other bacteria [41,42]. Third, Saccharibacteria-host cocultures could be passaged reliably in broth culture, but the Saccharibacteria component of the coculture was frequently lost by transfer on solid medium [3,42]. Fourth, other investigators’ successful or unsuccessful attempts at Saccharibacteria isolation suggested potential hosts: namely **Schaalia* odontolyticus*, host of TM7x [3]; *Leptotrichia* sp., comprising 50% of the reads in sequence for the ‘single cell isolate’ TM7c [14]; *Actinomyces graevenitzii* in the mixed sequence of a culture from the Karolinska Institute that contained Saccharibacteria HMT-352 (sequences communicated to FE Dewhirst, 21 August 2012, by Ovvind Kommmedal, Isentio AS, Paradis, Norway); and *Lachnoanaerobaculum saburreum* the filamentous bacterium isolated by Soro *et al*. that was thought to be TM7 [43]. These findings suggested that Saccharibacteria hosts may include bacteria from Gram-positive phyla Actinobacteria and Firmicutes, as well as the Gram-negative phylum Fusobacteria. Therefore, initial attempts to isolate human oral Saccharibacteria involved trying to infect strains of species from these phyla (Table 1, Study 1). Of the 11 initial potential hosts examined by adding Saccharibacteria to broth cultures, only *Arachnia propionica* (previously named *Pseudopropionibacterium propionicum*) [44,45] produced binary cocultures. The first two isolates, HB001 and AC001 were identified as Saccharibacteria bacterium HMT-488 and were isolated in culture with *A. propionica* strains F0230a and F0700, respectively. These cocultures could be stably passaged for months by taking 0.2 mL of old culture and adding it to 2.0 mL of fresh medium every 2 days (1:11 dilution). The third isolate, PM004 was identified as Saccharibacteria bacterium HMT-955 in coculture with *A. propionica* strain F0700. Unlike previous isolates TM7x, HB001 and AC001, PM004 appeared to kill its host and was passaged by 1:11 dilution into medium containing fresh host. These first three isolations validated the concepts that: Saccharibacteria could be isolated in coculture with an actinobacterial host, *A. propionica*, the coculture could be passaged in broth, and Saccharibacteria cells from Saccharibacteria containing clinical samples or cultures could be passed through a 0.2-micron membrane filter to remove other bacteria and retain infectivity. Table 3 provides full information on strains isolated including: subject from which isolate was obtained, host bacterial species and host strain, genome accession number, isolation method and isolation site. The genome sequences for Saccharibacteria bacterium HMT-488 strain AC001 and Saccharibacteria bacterium HMT-955 strain PM004 and a preliminary description of their isolation were recently reported [25,27,28]. In subsequent Studies 2 through 4, previously validated hosts or additional novel hosts were identified as indicated in Table 1.

**Table 3.** Saccharibacteria isolates.

Study	Subject series	Isolate species	Isolate taxon	Isolate strain	Strain Comment	Subject	Host species	Host taxon	Host strain	Genome	Isolation method	Site
1	1	Saccharibacteria bacterium	488	AC001	novel	UC19	*Arachnia propionica*	739	F0700	CP040003	direct filtration	Supragingival
1	1	Saccharibacteria bacterium	488	HB001	novel	UC19	*Arachnia propionica*	739	F0230	strain lost	filtered enrichment	Supragingival
1	1	Saccharibacteria bacterium	955	PM004	novel	UC16	*Arachnia propionica*	739	F0700	CP040008	direct filtration	Supragingival
2	1	Saccharibacteria bacterium	488	CM001	novel	UC15	*Arachnia propionica*	739	F0700	CP039999	direct filtration	Subgingival
2	1	Saccharibacteria bacterium	488	CM002	novel	UC03	*Arachnia propionica*	739	F0700	CP039998	direct filtration	Subgingival
2	1	Saccharibacteria bacterium	955	CM003	novel	UC01	*Arachnia propionica*	739	F0700	CP040010	filtered enrichment	Subgingival
2	1	Saccharibacteria bacterium	955	CM004	duplicate CM003	UC01	*Arachnia propionica*	739	F0700	none duplicate	direct filtration	Subgingival
2	1	Saccharibacteria bacterium	488	CM005	duplicate CM006	UC02	*Arachnia propionica*	739	F0700	none duplicate	direct filtration	Tonsils
2	1	Saccharibacteria bacterium	488	CM006	novel	UC02	*Arachnia propionica*	739	F0700	CP040001	direct filtration	Supragingival
2	1	Saccharibacteria bacterium	488	CM007	duplicate CM006	UC02	*Arachnia propionica*	739	F0700	none duplicate	filtered enrichment	Supragingival
2	1	Saccharibacteria bacterium	488	CM008	duplicate CM006	UC02	*Arachnia propionica*	739	F0700	none duplicate	direct filtration	Subgingival
2	1	Saccharibacteria bacterium	955	CM009	duplicate PM004	UC16	*Arachnia propionica*	739	F0700	CP040009	filtered enrichment	Subgingival
2	1	Saccharibacteria bacterium	488	CM010	novel	UC16	*Arachnia propionica*	739	F0700	CP039997	direct filtration	Subgingival
3	2	Saccharibacteria bacterium	488	FS03P	novel	FS-03	*Arachnia propionica*	739	F0700	CP040000	direct filtration	Supragingival
3	2	Saccharibacteria bacterium	952	FS04A	novel	FS-04	*Schaalia meyeri*	671	W712	in progress	direct filtration	Supragingival
3	2	Saccharibacteria bacterium	488	FS04P	novel	FS-04	*Arachnia propionica*	739	F0700	strain lost	direct filtration	Supragingival
3	2	Saccharibacteria bacterium	955	FS05P-A	novel	FS-05	*Arachnia propionica*	739	F0700	in progress	direct filtration	Supragingival
3	2	Saccharibacteria bacterium	488	FS05P-B	novel	FS-05	*Arachnia propionica*	739	F0700	CP047921	direct filtration	Supragingival
3	2	Saccharibacteria bacterium	952	FS06A	novel	FS-06	*Schaalia meyeri*	671	W712	in progress	direct filtration	Supragingival
3	2	Saccharibacteria bacterium	955	FS06P-A	novel	FS-06	*Arachnia propionica*	739	F0700	strain lost	direct filtration	Supragingival
3	2	Saccharibacteria bacterium	955	FS06P-B	novel	FS-06	*Arachnia propionica*	739	F0700	strain lost	direct filtration	Supragingival
3	2	Saccharibacteria bacterium	952	FS07A	novel	FS-07	*Schaalia meyeri*	671	W712	in progress	direct filtration	Supragingival
3	2	Saccharibacteria bacterium	488	FS07P	novel	FS-07	*Arachnia propionica*	739	F0700	CP047920	direct filtration	Supragingival
3	2	Saccharibacteria bacterium	952	FS09A	novel	FS-09	*Schaalia meyeri*	671	W712	in progress	direct filtration	Supragingival
3	2	Saccharibacteria bacterium	488	FS09P	novel	FS-09	*Arachnia propionica*	739	F0700	in progress	direct filtration	Supragingival
3	2	Saccharibacteria bacterium	952	FS10A	novel	FS-10	*Schaalia meyeri*	671	W712	strain lost	direct filtration	Supragingival
3	2	Saccharibacteria bacterium	488	FS13P	novel	FS-13	*Arachnia propionica*	739	F0700	CP047919	direct filtration	Supragingival
3	2	Saccharibacteria bacterium	955	FS14P	novel	FS-14	*Arachnia propionica*	739	F0700	CP047918	direct filtration	Supragingival
3	2	Saccharibacteria bacterium	488	FS15P	novel	FS-15	*Arachnia propionica*	739	F0700	CP047917	direct filtration	Supragingival
3	2	Saccharibacteria bacterium	955	FS17P	novel	FS-17	*Arachnia propionica*	739	F0700	CP047916	direct filtration	Supragingival
4	1	Saccharibacteria bacterium	488	AC002	reisolate HB001	UC19	*Arachnia propionica*	739	F0700	CP040002	direct filtration	Supragingival
4	1	Saccharibacteria bacterium	349	PM007	novel	UC19	*Actinomyces sp.*	897	F0631	in progress	direct filtration	Supragingival

Partial results from study 1 have been previously published [25]. Genomes for strains marked ‘in progress’ have been delayed due to temporary COVID-19 closure of the sequencing lab. When sequences are completed and annotated by NCBI they will be available within BioProject PRJNA282954 under strain isolate designation.

Following the successful isolation of these Saccharibacteria strains on the host *A. propionica*, we examined two variants of our isolation protocol on samples from five additional subjects. Information for these isolates is provided in Table 3 Study 2. Based on baseline subject-site microbiome results, one or more site samples were obtained from each of the five subjects. The host bacterium for these experiments was *A. propionica* strain F0700. Each sample was vortexed, divided into two aliquots, and one aliquot was immediately filtered and inoculated onto host in broth culture. The second aliquot was added directly to a host culture, grown for 7 days, and then filtered and inoculated into a fresh host broth culture. As shown in Table 3, 70% of the successful cocultures, CM001-CM010, were from the direct filtration protocol, and only 30% were from the filtered enrichment protocol involving inoculation, 4-day growth, filtration, and re-inoculation. While we initially hypothesized that adding host prior to filtration might expand the number of Saccharibacteria cells available for filtration, it appears this was not the case as fast-growing bacteria may have crowded out or inhibited Saccharibacteria expansion on its potential host. Based on these results, subsequent isolations used the direct filtration and inoculation protocol for isolation of Saccharibacteria with a host bacterium.

A third round of human oral Saccharibacteria isolation attempts used self-sampled plaque from 14 Forsyth Symposium attendees, Study 3. The samples from each subject were filtered, centrifuged, re-suspended and divided into 3 aliquots. The aliquots were inoculated into broth cultures of 3 host species previously shown to yield Saccharibacteria cocultures: namely *Arachnia propionica* HMT-739 strain F700, *Schaalia meyeri*, HMT-671 strain W712, and *Actinomyces* sp. HMT-170 strain F0386. Note several *Actinomyces* species have recently been moved to the genus *Schaalia* [46]. The results from this study are presented in Table 3, Study 3. Seventeen Saccharibacteria strains were isolated from 11 of the 14 subjects. This high isolation rate indicates that human oral Saccharibacteria can be isolated from most adults using this protocol. This high success rate arose despite failure to recover any cultures on host *Actinomyces* sp. HMT-170 strain F0386 (previously validated as a host for Saccharibacteria HMT-957 strain BB001 [25]. Isolates representing two distinct Saccharibacteria taxa growing on two different host species from the same subject sample inocula were seen for subjects FS04, FS06, FS07, and FS09 Table 3.

Isolation of Saccharibacteria in coculture with host revealed an unanticipated phenomenon. Saccharibacteria belonging to two different species were found to simultaneously infect a single host (a trinary culture) such as FS05P-A & FS05P-B and FS06P-A & FS06P-B (Table 3). Trinary cultures were identified for initial isolations from two subjects on the host *A. propionica*. This trinary infection was also seen in the earlier isolation of PM004 where the initial isolation showed an infection of the host with PM004 strain HMT-955 along with very low level of second Saccharibacteria HMT-488 strain. This low-level presence of a second Saccharibacteria species was identified during PacBio genome sequencing where in addition to the high coverage single contig for PM004, HMT-955, there were approximately 12 low coverage contigs that had high homology to genomes sequences for Saccharibacteria HMT-488 strains AC001 and HB001. The separation of both types of mixed cultures into pure binaries is described below. In a separate sampling of the subject (UC16), from whom PM004 had been isolated, the previously cryptic HMT-488 strain was isolated as CM010. The full genome sequence was 100% identical to the 12 contigs of partial sequence previously obtained.

In the course of getting several Saccharibacteria isolates into pure binary culture with their hosts, initial cultures were found to be contaminated with other bacteria about one third of the time. The most frequent contaminants were small motile organisms from the genera *Campylobacter* and *Capnocytophaga*. Contamination was observed by microscopy and/or plating on solid medium and obtaining 16S rRNA sequences for suspect colonies. Contaminations were successfully eliminated by one to three rounds of filtration and re-inoculation on fresh host. Additionally, binary cultures occasionally became contaminated during the many passages of long-term culture. Human skin species *Cutibacterium acnes, Staphylococcus aureus*, or *Staphylococcus epidermidis* were the most common passage contaminant, likely from human epithelial cells. Such contamination was easily eliminated by one round of filtration and re-inoculation on host.

In the three cases of ternary cocultures, a different approach was used to obtain pure binary cocultures. In each of these cases, cultures contained a Saccharibacteria HMT-488 and a Saccharibacteria HMT-955. As described in PCR methods and primers listed in Table 2, probe pairs specific for each species were created so that PCR of cultures or colonies could rapidly tell if a sample had Saccharibacteria HMT-488, HMT-955, both or neither. Samples from dual-infected cultures were plated on TSA plates at a dilution to yield about 100 colonies/plate. Approximately 100 entire colonies were picked and transferred into broth with fresh host. One µL of the culture was added to PCR reactions for the two the Saccharibacteria species. While >90% of colonies were negative for either Saccharibacteria (a phenomenon discussed further below), the rare positive led to the establishment of a pure binary culture. The difficulty in recovery of both Saccharibacteria species was in recovering less abundant species. This effort could require screening of several hundred colonies to identify one with the less abundant taxa. These observations show that a single host species can be infected with multiple Saccharibacteria species simultaneously in the same culture. Trinary cultures of *A. propionica*, Saccharibacteria HMT-488 and Saccharibacteria HMT-955 were stably passed into fresh host for at least 100 passages without loss of either Saccharibacteria species.

The fourth round of isolation attempts, Study 4, used self-sampling subjects as well as subjects from the initial 35-subject pool. The focus of this Study was to explore additional potential bacterial hosts, including taxa described by Podar’s laboratory such as *Actinomyces* sp. HMT-897 [47]. In work performed in our laboratory in 2015, Murugkar isolated the previously uncultured *Actinomyces* sp. HMT-897, strain F0631, and its genome was released in March 2018 (CP027236). Using strain F0631 as a potential host, Saccharibacteria bacterium HMT-349, strain PM007 was isolated. This isolate represents the fourth Saccharibacteria species isolated by our group. Genome sequencing of strain PM007 is in progress and will be associated with BioProject PRJNA282954 when available.

### Genomic information and strain fingerprinting

Currently we have closed PacBio genomes for 16 of the 32 strains isolated as indicated in Table 3. Sequencing is in process for an additional 7 unique strains, but genome sequencing has not been performed on 6 strains that are duplicates (isolated by different methods from the same subject) or 4 strains that were lost after preliminary fingerprinting information was obtained. The sequences of the genomes in progress will be accessible through their association with BioProject PRJNA28295.

**Figure 1.**
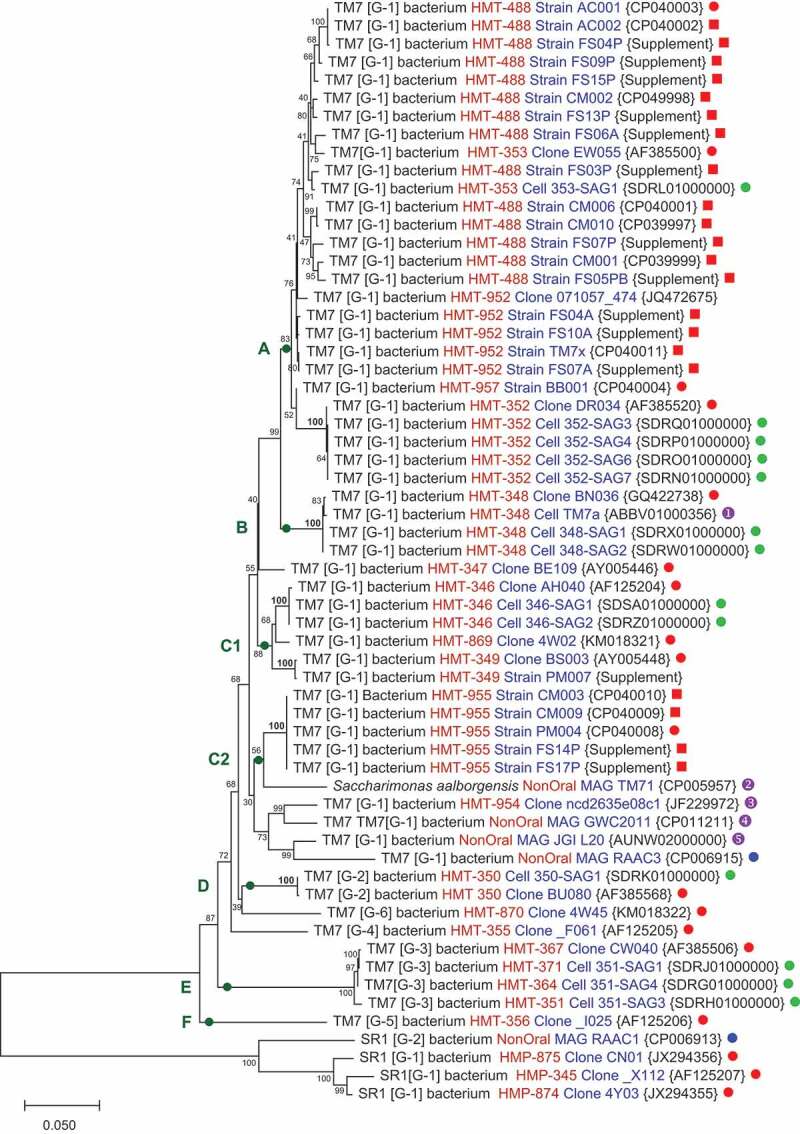
A 16S rRNA phylogenetic tree of human oral Saccharibacteria generated using neighbor joining analysis. The evolutionary distances were computed using the Jukes-Cantor method [72] and the scale bar represents substitutions per site. Bootstrap values are shown adjacent to each node and were calculated based on 500 replications. Square brackets indicate Saccharibacteria class level clades [26]. HMT-000 designates Human Microbial Taxon number in the Human Oral Microbiome Database [39]. GenBank accession numbers are in curly brackets. Source of sequences marked is indicated with following symbols: 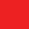
this work; 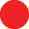
Previous Dewhirst lab publications [25,26,30] or GenBank deposits; 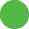
Cross *et al*. [47]; 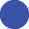
Kantor *et al*. [18]; 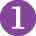
Marcy *et al*. [14]; 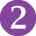
Albertson *et al*. [19]; 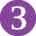
Kong *et al*. [73]; 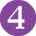
Brown *et al*. [20]; 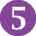
Tringe *et al*. (GenBank). Nodes to be compared between Figures 2–4 are marked with green circles and lettered A-F. The aligned sequences used to construct this figure can be found in Supplemental FASTA File-1.

**Figure 2.**
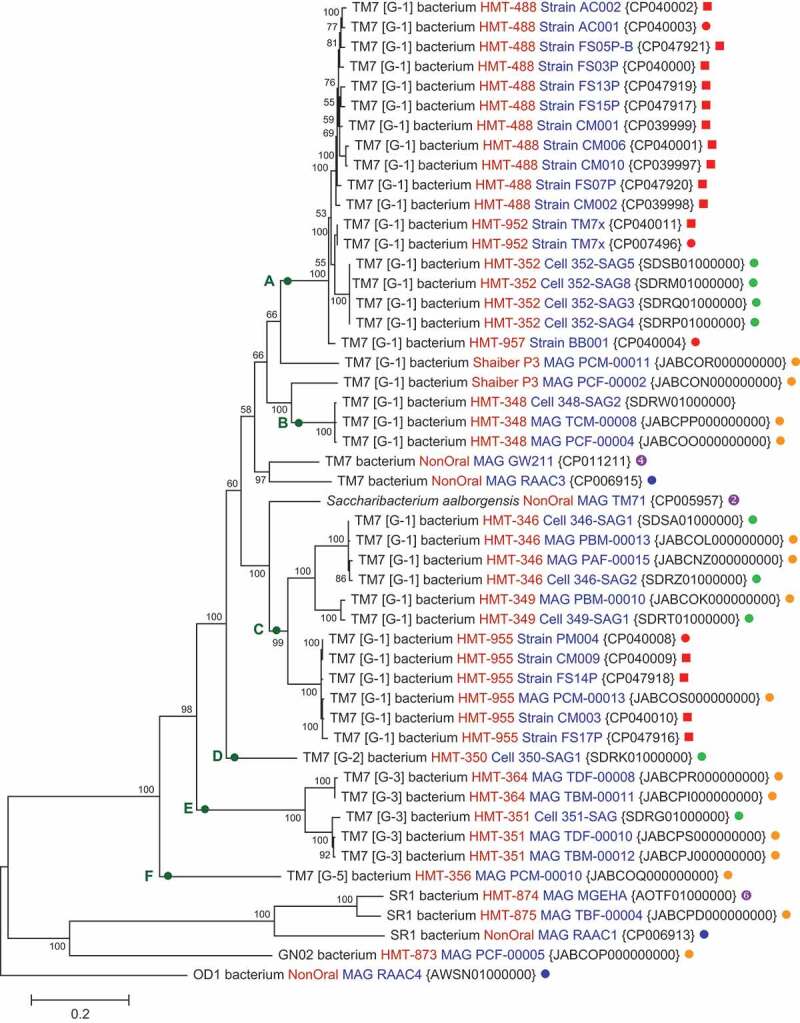
A phylogenomic tree bases on 27 concatenated ribosomal RNA proteins. The tree is a maximum likelihood tree where evolutionary distances were computed in units of number of amino acid substitutions per site. Bootstrap values were based on 1000 replicates. The tree was rooted and formatted in MEGA X [31]. Designations are as in Figure 1 except for source of sequences: 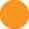
Shaiber *et al*. [34]. 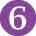
Campbell *et al*. [17].

**Figure 3.**
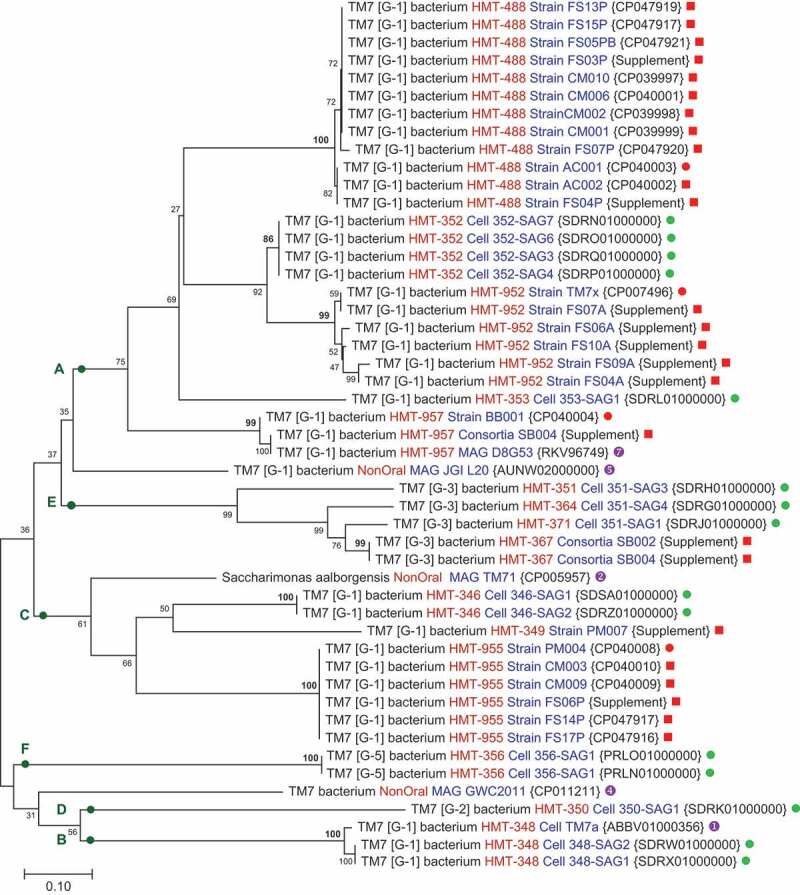
Phylogenetic tree of human oral Saccharibacteria based on analysis of a conserved 79 amino acid protein located in the ribosomal RNA operon between the 16S rRNA and the tRNA Ala (anticodon TGC). The evolutionary history was inferred using the Neighbor-Joining method [32]. The evolutionary distances were computed using the Poisson correction method [38] and are in the units of the number of amino acid substitutions per site. Evolutionary analyses were conducted in MEGA X [31]. Bootstrap values are shown adjacent to each node and were calculated based on 500 replications. Designations are as in Figure 1 except sequence source 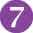
Espinoza *et al*. [74]. The aligned sequences used to construct this figure can be found in Supplemental FASTA File-2.

Previous studies of human oral Saccharibacteria species were based on analysis of single strains for most species and no more than two strains for any taxa [48]. Now with several isolates for Saccharibacteria HMT-952, HMT-488 and HMT-955, we can begin to examine the sequence diversity of strains both within a species and between strains of neighboring species. Phylogenetic trees based on 16S rRNA sequence comparisons, conserved genome proteins and that for a 79 amino acid protein are presented in Figures 1–3.

A phylogenetic tree generated from 16S rRNA sequences (Figure 1) separates Saccharibacteria taxa into several major clades marked A-F. The strain sequences for some taxa, such as HMT-352 in clade A, HMT-348 in clade B, HMT-346 and HMT349 in clade C1, HMT-955 in clade C2 and HMT-350 in clade D cluster into tight taxon groups that have bootstrap values of 100. However, the strain sequence for some taxa are poorly resolved from other taxa, with bootstrap values below 80, particularly in clade A. Notice that taxon HMT-353 strain sequences fall among strain sequences for taxon HMT-488. Strains from taxa HMT-952 and HMT-957 are not clearly separated from taxa HMT-488 and HMT-353. In clade E, the four different taxa cluster tightly together.

A phylogenetic tree generated from concatenated ribosomal proteins (Figure 2), displays significantly better separation of oral Saccharibacteria taxa from one another with tight clustering of strains within taxa than the 16S rRNA tree. It separates clade A taxa HMT-352, HMT-952 and HMT-488 into distinct clades with bootstrap values of 100. Clade E taxa HMT-351 and HMT-364 are well separated from one another. Unfortunately, the genomes for a number of strains for taxa present the 16S rRNA tree did not have sufficient genome coverage of ribosomal proteins to be included in the genome tree. Using average nucleotide identity (ANI) for the 50 genomes in Figure 2, strains within each taxon had ANI values of greater than 90%, whereas the ANI of strains between taxa was less than 82%.

A tree generated from a 79 aa protein located within the ribosomal RNA operon produces a highly resolved tree at the species level, with very high similarity between strains of individual species, Figure 3. This protein in the ribosomal RNA operon is discussed further below. Clade A is separated into five extremely well-resolved taxa, each with bootstrap values of 99 to 100 except for HMT-352 which as a bootstrap value of 86. Sequencing the region between the 16S rRNA and the tRNA yields the sequence for the 79aa protein and facilitates rapid identification of strains or metagenome reads to specific Saccharibacteria species level taxa. The 79 aa protein is of unknown function and not found outside the phylum Saccharibacteria. While this short protein is useful for species identification, it is not useful for phylogenetic reconstruction as major clades A to F are disordered compared to the 16S rRNA and ribosomal protein genome trees.

**Figure 4.**
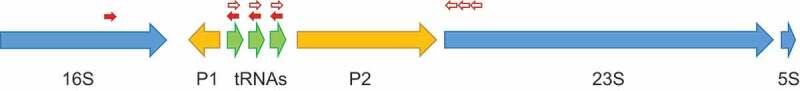
Ribosomal RNA operon structure for Saccharibacteria bacterium HMT-488 strain CM001. The ribosomal RNA genes are shown in blue. Transfer RNA genes are shown in green and ordered Ala (TGC), Ile (GAT) and Val (TAC). Proteins are shown in yellow. Protein P1 is a hypothetical protein which we designate the 79aa protein. Protein P2 is dihydroorate dehydrogenase (quinone) found in many oral Saccharibacteria at this position. In red are the PCR primers described in Table 2. Fingerprint primers 5–8 are shown as filled red symbols and are useful for obtaining the region from 16S rRNA to tRNA which includes the 79aa protein. Fingerprint primers 9–14 are shown in unfilled red symbols for amplifying the region from tRNAs to the 23S rRNA.

Authentication of key biological and chemical resources is a critical concern for scientific rigor and reproducibility. In microbiology, species are often identified by 16S rRNA sequence analysis. When one is working with multiple strains of a species, facile and reproducible strain identification tools become a necessity to prevent laboratory strain mix-ups. The gold standard for strain identification is a full genome sequence. For the many strains described in this report, full genome sequences are available (Table 3). However, a simpler identification scheme was needed for routine laboratory identification and for those strains currently without genome sequences. While 16S rRNA sequences are often identical for many strains within a species, the intervening region between 16S rRNA and 23S rRNA has been found useful for fingerprinting strains of many species [49,50]. This region is particularly valuable for fingerprinting species of Saccharibacteria as the ribosomal operon usually contains tRNAs and proteins between the 16S rRNA and 23S rRNA genes. For most all oral Saccharibacteria taxa, the region contains tRNA Ala (anticodon TGC), tRNA Ile (anticodon GAT) and tRNA Val (anticodon TAC) and coding regions for one to five proteins in various orientations. The structure of the ribosomal operon for Saccharibacteria bacterium MHT-488 strain CM001 and primer sites for fingerprinting are shown in Figure 4. Amplification using the forward 16S rRNA primer AI73 (Table 2) with the reverse primers for each of the three tRNAs (AI78-AI90) produced amplicon fingerprints (the order of the tRNAs is variable, so one chooses the amplicon with the longest coverage for sequencing) that distinguished each strain of each species from other strains of the species. For example, in Saccharibacteria bacterium HMT-488, strains FS15P and FS04P differ by 39 bases in the IVS region from the end of the 16S rRNA to the end of tRNA Ile. However, there are exceptions. The two highly similar strains HB001 and AC001, which were isolated from the same individual approximately 6 months apart, differed by 1 base in the 16S rRNA-tRNA fingerprint region. Using full genome comparisons of these two strains, a region was identified containing the ribosomal RNA small subunit methyltransferase A gene that allowed reliable distinction of strains (strains differ by 81 mismatches over a 601-base amplicon). Similarly, primers were designed to differentiate two closely related *Arachnia propionica* host strains (strains F0230 and F0700, Table 2). Because of the ease of mixing up strains of the same species, reliable fingerprinting is a necessity for regular authentication of bacterial strains that are key biological and chemical resources.

### Saccharibacteria stability, storage and revival

While Saccharibacteria species cocultured on *Actinomyces* hosts could be routinely revived from frozen glycerol stocks, Saccharibacteria on *A. propionica* hosts could not be revived. Testing *A. propionica* strain F0700 in growth media with a serial dilution of Glycerol (5%, 1%, 0.5%, 0.1%, 0.05%, 0.01%, 0.005%, and 0.001%) showed that growth was severely inhibited by glycerol at concentrations as low as 0.005%. Two other oral isolates of *A. propionica*, strains F0230a & F0236, were also inhibited by these low concentrations of glycerol. It appears that glycerol is sufficiently toxic to *A. propionica*, which even with addition of fresh host and stocks diluted to >0.005% glycerol, Saccharibacteria–host cocultures could not be revived. The mechanism of toxicity is unknown. After losing all glycerol stocks of Saccharibacteria cocultured with *A. propionica*, subsequent stocks were preserved in 5% DMSO solution and these stocks were reliably revived after freezing at minus 80°C.

Many organisms that are difficult to cultivate are fastidious, and highly oxygen sensitive, for example species in the genus *Treponema*. Saccharibacteria HMT-488 strain AC001 and HMT-955 strain PM004 were cocultured with *A. propionica* for over 10 passages under aerobic, microaerophilic and anaerobic conditions. Thus, these Saccharibacteria are not inhibited by atmospheres of up to 20% oxygen.

## Discussion

### Isolation and culture protocol

**Figure 5.**
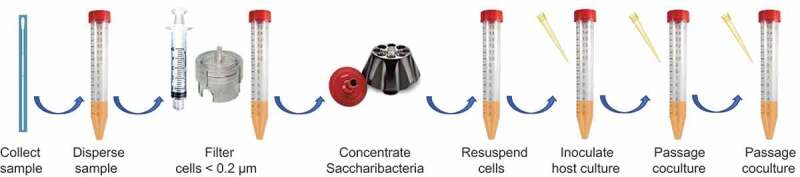
Human oral Saccharibacteria isolation and culture methods. Oral samples are dispersed and applied to a 0.2 µm membrane filter which allows passage on only small cells. The filtered cells are concentrated by ultracentrifugation and resuspension in a small volume. An aliquot is inoculated into a broth culture of host cells to establish a Saccharibacteria-host coculture. The coculture is then passaged by dilution into fresh medium.

Based on the studies reported here, a recommended an optimized seven step method for the isolation and culture of human oral Saccharibacteria is presented in Figure 5. Clinical samples are collected using swab, brush or curette and dispersed into 1 mL MRD buffer and vigorously vortexed. The sample is diluted with 9 mL MDR buffer and filtered through a 47 mm 0.2-micron track-etched polycarbonate filter held in a 47 mm filter holder. The filtrate is concentrated by ultracentrifugation and the Saccharibacteria pellet is resuspended in 1 mL MRD buffer by vortexing. An aliquot (100 µL to 300 µL) of the resuspended Saccharibacteria cells are added to a 2 mL culture of one or more host bacteria in broth. The initial inoculated host cultures are incubated for 5 days to allow infection to develop. Cultures are passaged every 3 days thereafter by transfer of 200 µL of coculture to 2 mL of fresh medium or to 2 mL fresh medium containing host cells if Saccharibacteria strain kills host. This protocol should be adaptable to other human body sites, to other animal species and potentially environmental sites where an adequate number of cultured potential host species are available.

### Conceptual and methodological roadblocks and issues in literature

There are many reasons why it took nearly 20 years from the discovery of TM7 (Saccharibacteria) and other CPR bacteria until Saccharibacteria isolates were successfully cultured. We will discuss the conceptual and methodological roadblocks that had to be overcome to permit our successful isolation of 32 Saccharibacteria strains so that other investigators can avoid these roadblocks in their attempts to culture other members of the CPR.

### Parasites need hosts

The first roadblock was the assumption that Saccharibacteria were simply fastidious bacteria that were capable of axenic growth in a complex medium under the proper culture conditions. We now know that all Saccharibacteria isolated to date are obligate parasites that require the presence of live bacterial hosts. Thus, anyone using Koch’s approach of streaking for isolation and axenic culture was doomed to fail because it excludes the necessary bacterial host. The idea that obligate parasitic organisms require a host is an old concept in biology, but possibly under-appreciated by the microbiologists attempting to culture candidate phyla bacteria before the isolation of TM7x. It is well recognized that there are bacterial parasites of eukaryotic cells such as members of the genera *Chlamydia* and *Mycoplasma*, the insect endosymbiont *Buchnera* spp [51], and *Vampirovibrio chlorellavorus*, a small, 0.6-micron diameter, epi-parasite of the green alga *Chorella vulgaris* [52]. However, awareness of the fact that bacteria parasitize other bacteria was generally overlooked except for *Bdellovibrio bacteriovorus* and related bacteria [53]. Therefore, despite solid literature on specific prokaryotes being parasites on other prokaryotes, there are no reports that microbiologists attempted to culture CPR bacteria in binary coculture before the isolation of TM7x. It appears that most investigators attempted to obtain pure cultures directly following Koch’s approach.

### Passage of host-parasite cocultures

The second was the assumption that members of a Saccharibacteria consortium could be passaged on plates. We now know that Saccharibacteria/host cocultures can be easily passaged in broth culture, but when transferred on plates, only uninfected host cells grow to form colonies (only one in 50 to 100 colonies are infected with Saccharibacteria, possibly due to an uninfected colony overgrowing a Saccharibacteria cell). Therefore, passage on plates almost always results in loss of the Saccharibacteria component and to a pure culture of only host cells. The utility of plate culture in the isolation and propagation of microbes is so ingrained in microbiological thinking and practice, that to exclude its use in the isolation and culture of Saccharibacteria and other CPR bacteria requires a break from the standard microbiological paradigms.

### Fastidiousness/auxotrophy

A third misconception about Saccharibacteria that may apply to other uncultured bacteria, including other CPR phyla, is that the uncultured status corresponds with fastidiousness and fragility. For example, many *Treponema* spp. are fastidious oxygen-sensitive obligate anaerobes that are fragile to mechanical manipulation. The Saccharibacteria we have isolated, in contrast, can be grown aerobically, microaerophilically or anaerobically depending only on their host’s oxic range and survive filtration and ultracentrifugation. Culture of Saccharibacteria required the presence of a host bacteria, not addition of novel factors to standard microbial media. Rather than varying media and conditions to achieve growth, binary or ternary bacterial coculture may fulfill all auxotrophic needs of some difficult to culture organisms.

### Size and morphology

The fourth misconception involves the perceived size and morphology of Saccharibacteria and the use of fluorescent in situ hybridization (FISH) for their identification. There is a substantial literature which suggested that Saccharibacteria are large rods. In a seminal paper describing the Candidate Division TM7, Hugenholtz et al [2], presented FISH and electron microscopy images of cells that they identified as TM7s. The FISH images showed both cocci and long filamentous organisms. The electron micrographs showed sheathed filamentous bacteria. Based on this work, many TM7 investigators (including the authors of this manuscript) assumed that TM7s were large filamentous bacteria.

The assumption that TM7s were large filamentous rods was further strengthened by the pioneering work of Relman’s group using FISH-stained cells for single cell isolation [54]. Relman’s group targeted filamentous cells for single cell isolation and obtained genome sequences for single cell isolates TM7a, TM7b and TM7c [14]. The filamentous nature of TM7s seemed validated with the description of the axenic culture of a candidate division TM7 bacterium from the human oral cavity [43]. Using FISH methods, Soro *et al*. isolated a filamentous organism that was initially identified by PCR as TM7 bacterium HMT-356.

However, we believe the assumptions and conclusions that TM7s are large filamentous bacteria were mistaken. The most complete TM7 genome of Marcy *et al*., TM7c, (ABBX01000000.1) contained 129 contigs of total length 0.474 Mbp. The contigs were composed of equal parts Saccharibacteria HMT-348 and *Leptotrichia* sp. HMT-417 related to *Leptotrichia wadeii*. Human oral *Leptotrichia* species are known to be long rods [55,56] and thus the Marcy *et al*. findings are consistent with them having isolated a single cell long rod *Leptotrichia* sp. cell to which Saccharibacteria HMT-348 cells were attached. Following publication of the Soro *et al*. paper [43], the authors kindly provided their isolate to the Dewhirst laboratory for study. The 16S rRNA sequence obtained for the strain received was identified as being a pure culture of the filaments bacterium *Lachnoanaerobaculum saburreum* [57]. No Saccharibacteria cells or DNA were detected by TM7 taxon HMT-356 specific PCR in the culture provided. Subsequently, the Jenkinson laboratory examined their stock cultures and could not find one that was PCR positive for the Saccharibacteria present originally. Thus, it appears they had a TM7 in coculture with *Lachnoanaerobaculum saburreum* (and possibly other bacteria), but through purification on plates they ended up losing the TM7 component and getting a pure culture of a long rod-shaped bacterium.

The identification of target organisms in a consortium using FISH probes is technically difficult [58], particularly when trying to identify an organism of unknown size and shape and for which one has no reference organisms with which to adjust and validate probe hybridization conditions. Issues with FISH probes for TM7 identification were noted by Slava Epstein’s group [59]. They found that several published FISH probes and PCR primers for TM7 bacteria cross-reacted with or amplified DNA from several oral and vaginal filamentous bacteria. Rod and filament morphology are also common in environmental samples and include members of the phylum Chloroflexi [60] and the genera *Sphaerotilus* [61] and *Leptothrix* [62] from the phylum Proteobacteria. Since isolated Saccharibacteria are just at the limit of resolution using light microscopy, it is problematic to develop and validate FISH probes for environmental Saccharibacteria if they are similarly small. The fluorescence signal from ~0.2 μm diameter cells could appear to come from much larger host cells if viewed using a broad field of view. Now that cocultures with live Saccharibacteria are available for study, it should be easier for laboratories to optimize their hybridization conditions for FISH probes.

### Evidence for Saccharibacteria and CFP bacteria being small cells

**Figure 6.**
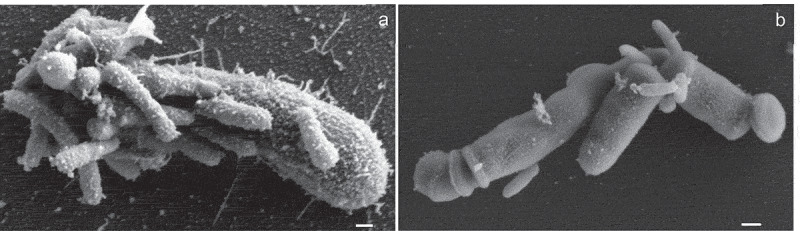
Scanning Electron Micrographs of *Saccharibacteria* infected *Arachnia propionica*. Cocultures of *Saccharibacteria* strains AC001 (a) and PM004 (b) with *P. propionicum* were observed under SEM. The size difference between the host and *Saccharibacteria* cells was evident. Multiple cells of *Saccharibacteria* were attached to single host cell, Scale bar: 100 nm.

Each of the 32 Saccharibacteria we have isolated has been passed through a 0.2 µm membrane filter. Those visualized by microscopy are cocci <0.2 um in diameter or coccobacilli with diameter of 0.1–0.2 µm. The small size is also evident in Scanning Electron Micrographs (SEM) that our lab generated (Figure 6).While not excluding the possibility that some Saccharibacteria or members of other CPR phyla are larger than the human oral Saccharibacteria isolates, for sake of argument, we hypothesize there are none. This hypothesis can be convincingly falsified by investigators isolating larger CPR bacteria (in coculture or stable consortia), demonstrating that the isolates can be stably passaged in long-term culture and by making these larger bacterial isolates available to other investigators for verification.

The first indication that members of CPR phyla were physically small in size came from the 2005 paper by Miyoshi *et al*., who characterized 16S rRNA gene clones from deep-groundwater that pass through 0.2-micron-pore-size filters [63]. They obtained many clone sequences from the OD1 (Parcubacteria) and OP11 (Microgenomates) phyla. The first major paper to build on this observation was that of Luef *et al*., in 2015 [41]. This paper from the Banfield group presented 3D cryo-electron tomographic images of ultra-small bacteria that pass through 0.2- and 0.1-micron filters. The microorganisms were almost exclusively from the OD1 (Parcubacteria), OP11 (Microgenomates) and WWE3 (Katanobacteria) phyla. The cells examined had an average spherical diameter of 0.25 µm and volume of 0.008 µm^3^. Thus, at least some bacteria from these three CPR linages are ultra-small bacteria. The work of Luef *et al*., bridges current molecular studies back to older literature on ultramicrobacteria.

The preferred term for the smallest viable bacteria is ultramicrobacteria (UMB) [64]. There is a substantial literature on the theoretical minimum size of a bacterium, which grew out of an effort to refute claims of bacterial cells orders of magnitude smaller than 0.2 µm in diameter. The disputed papers were the purported bacterial fossils in Martian meteorite ALH84001 [65] and nanobacteria in pathogenic calcifications and stone formations [66]. Examples of cultivation of UMB cells with equivalent diameters of 0.2 to 0.4 µm and volumes of 0.004 to 0.034 µm^3^ include members of the well-known genera *Chlamydia* and *Mycoplasma*, isolates from urban soil [67], *Sphingopyxix alaskensis* [68], *Dialister invisis* from the human oral cavity [69], and *Nanoarchaeum equitans* [70]. The lower boundary of the UMB minimum size is reliably based on cultivated organisms. As CPR organisms are cultured, the minimum viable cell size will likely drop somewhat, but is thought that a volume of 0.002 µm^3^ (diameter 0.156 µm) is about the limit [41,71].

### Conclusions

We have successfully established an isolation approach that has allowed us to obtain 32 strains of human oral Saccharibacteria. The strains belong to four species of Saccharibacteria: namely HMT-349, HMT-488, HMT-952, HMT-955 and were isolated from 11/14 (78%) of the subjects sampled in our methods validation study. Approaches, ideas and methods that facilitate the isolation of Saccharibacteria require abandoning many of the standard paradigms of microbiology. It is hoped that the simple methods presented here will allow investigators to isolate strains of hearty and easy to culture human oral Saccharibacteria species in coculture with their bacterial hosts. It is hoped that the extensive discussion of conceptual issues will help investigators avoid the pitfalls of classic isolation and culture methods. These isolates can be studied in their own right or serve as positive controls for all manner of follow on bench research such as the validation of FISH probes in environmental settings. It is hoped that the approach to isolation and culture of human oral Saccharibacteria described here will be of benefit to the isolation of Saccharibacteria from other human body sites, mammalian hosts, environmental settings, and possibly to the isolation of representatives of other CPR phyla.

## Supplementary Material

Supplemental Material

## Data Availability

The genome sequences for Saccharibacteria are available from NCBI under accession numbers listed in Table 3 and under BioProject PRJNA282954. 16S rRNA and 79 amino acid protein sequences used to make Figures 1 and 3 that are not part of a deposited genome are available in Supplementary file 16S rRNA FASTA file 16S rRNA and Supplementary FASTA file 79aa.
